# The Myb domain of the largest subunit of SNAPc adopts different architectural configurations on U1 and U6 snRNA gene promoter sequences

**DOI:** 10.1093/nar/gku905

**Published:** 2014-10-16

**Authors:** Yoon Soon Kang, Michelle Kurano, William E. Stumph

**Affiliations:** 1 Department of Chemistry and Biochemistry, Molecular Biology Institute, San Diego State University, San Diego, CA 92182-1030, USA; 2 Department of Biology, Molecular Biology Institute, San Diego State University, San Diego, CA 92182-1030, USA

## Abstract

The small nuclear RNA (snRNA) activating protein complex (SNAPc) is essential for transcription of genes that encode the snRNAs. *Drosophila melanogaster* SNAPc (DmSNAPc) consists of three subunits (DmSNAP190, DmSNAP50 and DmSNAP43) that form a stable complex that recognizes an snRNA gene promoter element called the PSEA. Although all three subunits are required for sequence-specific DNA binding activity, only DmSNAP190 possesses a canonical DNA binding domain consisting of 4.5 tandem Myb repeats homologous to the Myb repeats in the DNA binding domain of the Myb oncoprotein. In this study, we use site-specific protein–DNA photo-cross-linking technology followed by site-specific protein cleavage to map domains of DmSNAP190 that interact with specific phosphate positions in the U6 PSEA. The results indicate that at least two DmSNAP190 Myb repeats contact the DNA in a significantly different manner when DmSNAPc binds to a U6 PSEA versus a U1 PSEA, even though the two PSEA sequences differ at only 5 of 21 nucleotide positions. The results are consistent with a model in which the specific DNA sequences of the U1 and U6 PSEAs differentially alter the conformation of DmSNAPc, leading to the subsequent recruitment of different RNA polymerases to the U1 and U6 gene promoters.

## INTRODUCTION

Most of the spliceosomal small nuclear RNAs (snRNAs) are synthesized by RNA polymerase II (Pol II), but U6 snRNA is synthesized by RNA polymerase III (Pol III) (1–9). Despite this difference in RNA polymerase specificity, transcription of both classes of genes relies upon a unique DNA binding transcription factor called the snRNA activating protein complex (SNAPc) (10,11) or proximal sequence element (PSE) binding transcription factor (PTF) (12,13). In the fruit fly *Drosophila melanogaster*, DmSNAPc consists of three subunits known as DmSNAP190, DmSNAP50 and DmSNAP43 (9,14–15). These three subunits are conserved throughout metazoan evolution, and homologous proteins are found even in the early-diverging trypanosomes (16–19). DmSNAPc recognizes a ∼21 bp promoter element termed the proximal sequence element A (PSEA) located ∼40 to 60 bp upstream of the transcription start site of both the Pol II- and Pol III-transcribed genes (5,9,20).

The PSEAs of the two best-studied fly snRNA genes (U1:95Ca and U6:96Ab) differ at only 5 out of their 21 nucleotide positions, yet these two PSEAs are not interchangeable. The sequence of the U1 PSEA is unable to promote transcription by Pol III, and the U6 PSEA cannot promote transcription by Pol II, even though both sequences are bound by DmSNAPc *in vitro* and *in vivo* and function effectively in the context of their own native genes. Transcription assays *in vitro* indicated that the specific nucleotide sequence of the PSEA was the dominant element for determining polymerase specificity in flies, as a switch in RNA polymerase specificity occurred when the U1 and U6 PSEAs were exchanged (5,21). Specifically, the bases at nucleotide positions 19 and 20 within the PSEA appeared to be the most important for determining RNA polymerase specificity in an *in vitro* transcription assay (5). In cellular transfection assays and in transgenic flies, switching the PSEAs resulted in a complete loss of gene expression (21,22).

Evolutionary evidence also supports the concept that specific nucleotide differences within the PSEAs are important for determining RNA polymerase specificity. When the *D. melanogaster* PSEAs of 23 Pol II snRNA genes and seven Pol III snRNA genes were compared, all 23 Pol II genes had a ‘G’ or an ‘A’ at position 19, whereas all seven Pol III genes had a ‘T’ at this position. Similarly, a ‘G’ was conserved at position 20 in the Pol II PSEAs, but a ‘C’ was conserved at that position in the Pol III PSEAs (9). Furthermore, in a bioinformatic study of Pol II and Pol III PSEAs from five other insect species, certain nucleotide positions in the 3′ half of each species’ PSEAs were ‘conserved-to-be-different’ between the Pol II- and Pol III-transcribed genes, although the specific positions where these differences occurred varied among the different species (20).

**Figure 1.**
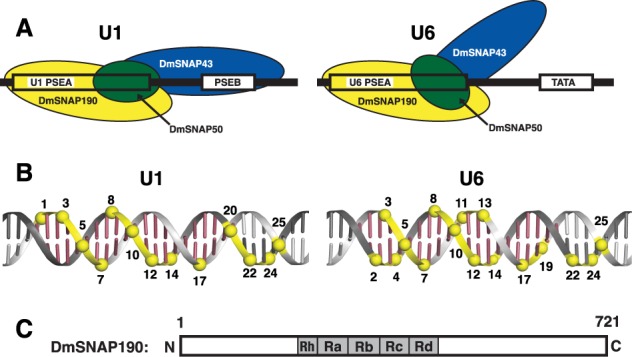
(**A**) Schematic drawing of DmSNAPc bound in different conformations to U1 and U6 snRNA gene promoters. *Drosophila* Pol II snRNA gene promoters consist of a PSEA and a PSEB with an 8 bp separation in between, but U6 promoters consist of a PSEA and a TATA box separated by 12 bp (20). The PSEB and TATA boxes affect the efficiency of transcription but not the choice of RNA polymerase (5). The DmSNAPc subunits are represented by yellow, green and blue ovals (DmSNAP190, DmSNAP50 and DmSNAP43 respectively). (**B**) Contact points between the DmSNAP190 subunit and the U1 PSEA or U6 PSEA based upon site-specific protein–DNA photo-cross-linking (14). Raspberry-colored bases indicate the 21 bp of the U1 and U6 PSEAs. The yellow spheres indicate phosphate positions that specifically cross-linked to DmSNAP190 as a subunit of DmSNAPc bound to DNA. (Only every second phosphate position was assayed on each strand. Odd-numbered phosphates are on the non-template strand and even-numbered phosphates are on the template strand.) (**C**) A schematic diagram of DmSNAP190 protein, which consists of 721 amino acid residues and 4.5 Myb repeats designated Rh (the half repeat), Ra, Rb, Rc and Rd.

Site-specific protein–DNA photo-cross-linking assays have revealed that all three subunits of DmSNAPc contact the DNA. In general terms, DmSNAP190 (the largest subunit) contacts the entire length of the PSEA whereas the two smaller subunits contact primarily the downstream half of the PSEA (14,15). Interestingly, the protein–DNA cross-linking studies also revealed that each subunit contacted the DNA differently depending upon whether DmSNAPc was bound to a U1 PSEA or to a U6 PSEA (14,15). This led us to propose a working model in which DmSNAPc adopts different conformations on U1 and U6 PSEAs (Figure 1A). We hypothesize that these conformational differences subsequently lead to recruitment of different general transcription factors and distinct RNA polymerases.

Figure 1B shows the actual patterns of DmSNAP190 cross-linking to U1 or U6 PSEA-containing DNAs. The yellow spheres indicate phosphate positions that cross-linked to DmSNAP190 when DmSNAPc was bound to a U1 PSEA (left duplex) or to a U6 PSEA (right duplex). In both cases, DmSNAP190 cross-linked over the entire length of the PSEA. Although the cross-linking patterns exhibited many similarities, in each case there were several specific phosphate positions where DmSNAP190 cross-linked to the U1 PSEA only or conversely to the U6 PSEA only. The differences in the cross-linking patterns of the two smaller subunits of DmSNAPc to the U1 and the U6 PSEAs were even more pronounced than observed with DmSNAP190 (illustrated schematically in Figure 1A) (14–15,23).

In recent work, we developed novel methodology in which we combined the site-specific protein–DNA photo-cross-linking assay with subsequent site-specific digestion of the protein (23). This allowed us to map sub-regions or domains of DmSNAP50 and DmSNAP43 that cross-linked to specific phosphate positions in U1 or U6 promoter DNA (23). A similar analysis was subsequently carried out that identified domains of DmSNAP190 that cross-linked to each of the 13 phosphate positions in the U1 PSEA to which DmSNAP190 was found to cross-link (Figure 1B) (23–25). However, similar work has not yet been reported for DmSNAP190 on a U6 PSEA.

The domain structure of DmSNAP190 is shown in Figure 1C. The most obvious feature of metazoan SNAP190 is that it contains 4.5 tandem Myb repeats, termed Rh, Ra, Rb, Rc and Rd (15,26). Myb repeats, each ∼50 amino acid residues in length, were first characterized in the Myb oncoprotein. The Myb protein itself contains three tandem Myb repeats that form its DNA binding domain (27–29). To our knowledge, SNAP190 is the only protein that contains more than three Myb repeats. The N-terminal and C-terminal domains of DmSNAP190 that flank the Myb domain are not known to share significant homology with any other proteins.

Here we report the results of site-specific protein–DNA photo-cross-linking coupled with site-specific protein digestion to localize domains of DmSNAP190 on the U6 PSEA. Overall, we found that the general orientation of DmSNAP190 on U1 and U6 promoter sequences is similar. However, we also found that there is a significant shift in the positions of at least two of the Myb repeats such that minor groove-spanning interactions take place on the U6 PSEA that are absent on the U1 PSEA. With these data, we are now able for the first time to draw a comprehensive picture of the conformational differences of the DmSNAP complex on U1 and U6 promoter sequences.

## MATERIALS AND METHODS

### DmSNAPc constructs, expression and purification

Untagged and N- or C-terminally FLAG-tagged constructs encoding DmSNAPc subunits have been previously described (23,25,30). Various DmSNAP190 constructs with single hydroxylamine cleavage sites (asparaginyl-glycyl (NG) peptide bonds) have also been previously described (23,25). By using the same methods, two new DmSNAP190 constructs were prepared for this work, each with a single NG peptide bond at residues 445–446 and 463–464 respectively.

DmSNAPc variants were over-expressed in *D. melanogaster* S2 cells stably co-transfected with DmSNAP190, DmSNAP50 and DmSNAP43 constructs each driven by the metallothionein promoter (23,25,30). DmSNAP190 was FLAG-tagged at its N-terminus except in those cases when untagged DmSNAP190 was desired. In the latter case, N-terminally FLAG-tagged DmSNAP43 was co-transfected. FLAG immunoaffinity chromatography was used as previously described as a purification step for the DmSNAP complexes (23,25,30). The DNA binding activity of each variant DmSNAPc was confirmed by electrophoretic mobility shift assays.

### Site-specific protein–DNA photo-cross-linking, protein hydroxylamine digestion and detection of cleaved fragments

Double-stranded DNA probes that each contained a photo-cross-linking agent (azidophenacyl group) located at a specific individual phosphate position within or downstream of the U6 PSEA were prepared exactly as described in detail previously for U1 probes (23,25). The azidophenacyl group was attached through a phosphorothioate, and each probe contained a ^32^P radiolabel at the second phosphate position 5′ of the cross-linker on the same DNA strand. Each double-stranded probe consisted of the following non-template strand DNA sequence, and its complement, with the cross-linker at one specific designated position in either the non-template or template DNA strand: 5′-GCTATGACCATGATTACGAATTCATTCTTA**TAATTCTCAACTGCTCTTTCC**GGTACCGCCATGGAAAGGTATGGGATC-3′. The bold nucleotides indicate the sequence of the 21 bp U6 PSEA from the U6:96Ab gene. The underlined nucleotides indicate the five positions where the U6 PSEA bases differ from the PSEA bases of the U1:95Ca gene used in previous photo-cross-linking studies (25). Outside of those five positions, the sequence of the U6 and U1 photo-cross-linking probes were identical to ensure that differences in U1/U6 cross-linking were due solely to the five nucleotide differences within the PSEA.

Protein–DNA binding, photo-cross-linking and hydroxylamine digestion of the cross-linked proteins were carried out exactly as previously described (23,25). Digested, radiolabeled protein fragments were identified by denaturing gel electrophoresis and autoradiography.

### Immunoblots of hydroxylamine-digested DmSNAPc

Detection of N-terminal and C-terminal fragments of DmSNAP190 variants by immunoblotting was carried out exactly as previously described (23,25). Anti-FLAG monoclonal antibody was used to detect N-terminal fragments specifically, and antibodies directed against a 14 amino acid peptide from the C-terminus of DmSNAP190 were used to detect C-terminal fragments.

## RESULTS

### Identification of DmSNAP190 Myb domain repeats that cross-link to U6 PSEA positions 3, 5 and 7 of the non-template DNA strand

As illustrated in Figure 1B, DmSNAP190 can be cross-linked to phosphate positions 3, 5 and 7 of the non-template strand of the U6 PSEA DNA. To localize the region of DmSNAP190 that cross-linked to each of these three positions, we performed site-specific protein–DNA photo-cross-linking by using FLAG-immunoaffinity-purified DmSNAPc and U6 DNA probes that contained a cross-linker at U6 PSEA positions 3, 5 or 7. We then carried out hydroxylamine cleavage of the protein and then detected and identified cross-linked protein fragments by gel electrophoresis and autoradiography.

**Figure 2.**
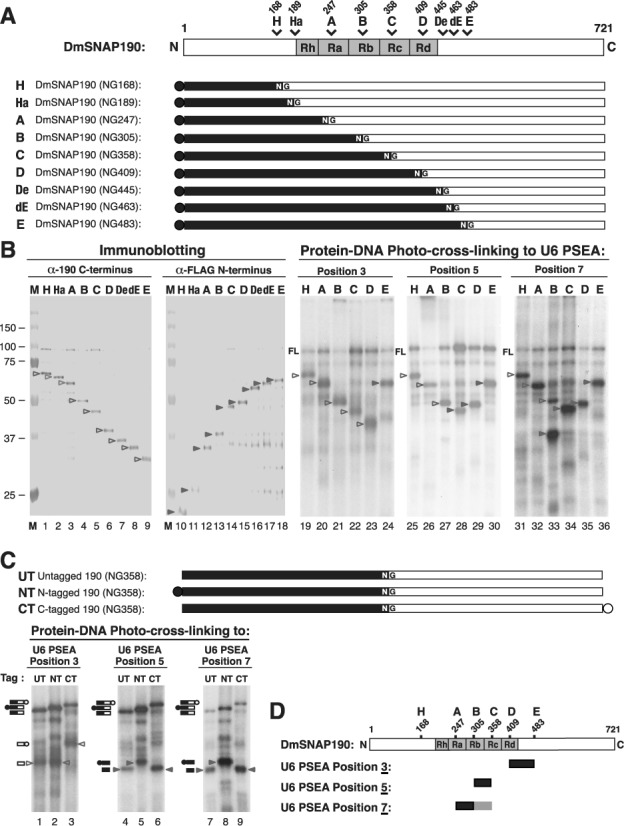
Mapping DmSNAP190 Myb repeat domains that cross-link to U6 PSEA positions 3, 5 and 7 of the non-template strand. (**A**) The top bar represents a schematic diagram of DmSNAP190 and shows the position of nine different hydroxylamine cleavage sites present individually in the nine different constructs represented below (constructs H through E). The darkly shaded and unshaded regions represent the N-terminal and C-terminal fragments expected to be produced from each construct by hydroxylamine digestion. (**B**) All gel results shown are subsequent to hydroxylamine digestion of DmSNAPc from cell lines separately expressing the DmSNAP190 variants represented by constructs H to E shown in (A) above. The first two panels show immunoblots using antibodies against a synthetic C-terminal peptide of DmSNAP190 (lanes 1–9) or antibodies against the FLAG epitope at the N-terminus of each construct (lanes 10–18). Lanes marked M contain molecular weight markers. Arrowheads (unshaded for C-terminal fragments and shaded for N-terminal fragments) point out the specific cleavage products of interest. The three panels to the right show autoradiography results of site-specific protein–DNA photo-cross-linking of the DmSNAP190 constructs H, A, B, C, D and E to U6 PSEA position 3 (lanes 19–24), position 5 (lanes 25–30) or position 7 (lanes 31–36). The label FL indicates the position of bands corresponding to full-length undigested N-tagged DmSNAP190. Arrowheads point out the bands corresponding to cross-linked C-terminal (unshaded) or N-terminal (shaded) fragments. In lane 33, both the N- and C-terminal fragments of construct B cross-linked to position 7. (**C**) The bars in the upper part of the figure represent three DmSNAP190 constructs (UT, NT and CT) that are untagged, N-tagged and C-tagged, respectively, and each contains a single NG cleavage site at position 358. Construct NT and construct C (from panel A) are actually identical but are labeled differently in different parts of the figure for clarity. The three panels below show the results of protein–DNA photo-cross-linking of the UT, NT and CT constructs to U6 PSEA position 3 (lanes 1–3), position 5 (lanes 4–6) or position 7 (lanes 7–9). Unshaded arrowheads indicate bands representing C-terminal fragments after hydroxylamine digestion, and shaded arrowheads point out N-terminal fragments. The symbols alongside the gel schematically indicate the origin of each band and correspond to the diagrams of the constructs above. (**D**) Schematic diagrams representing the region of DmSNAP190 that cross-linked to U6 PSEA positions 3, 5 or 7. Dark shading represents stronger cross-linking and lighter shading represents weaker cross-linking.

For different portions of this work, nine different DmSNAP190 constructs were utilized that each contained a single NG peptide bond that could be cleaved with hydroxylamine. The schematic diagram at the top of Figure 2A shows the position of the NG site within each protein construct (H through E). The locations of the 4.5 Myb repeats Rh, Ra, Rb, Rc and Rd, are also indicated. Each of the nine constructs contained a FLAG tag at its N-terminus. The N- and C-terminal fragments expected after hydroxylamine digestion of each construct are represented in the lower part of Figure 2A by the darkly shaded and the unshaded areas respectively.

Western blots of these protein constructs following hydroxylamine digestion are shown in the first two panels of Figure 2B (lanes 1–18). Lanes 1–9 show the results of detection with antibodies specific to the C-terminus. Fragments of decreasing size were observed that corresponded to the unshaded fragments shown in Figure 2A. Lanes 10–18 show the N-terminal fragments of increasing size (detected using anti-FLAG antibodies) that corresponded to the shaded fragments in Figure 2A.

The third panel of Figure 2B (lanes 19–24) shows an autoradiogram of ^32^P-labeled protein fragments run on a denaturing gel after hydroxylamine digestion of DmSNAP190 following site-specific cross-linking to position 3 of the U6 PSEA. The pattern of decreasing-size fragments in lanes 19–23 is consistent with the cross-linking of phosphate position 3 to the C-terminal DmSNAP190 fragment from each of the constructs H through D. In contrast, phosphate position 3 cross-linked to the N-terminal fragment of construct E (lane 24). These results, taken together, indicate that phosphate position 3 cross-linked to DmSNAP190 C-terminal of cut site D but N-terminal of cut site E. We therefore can conclude that a domain of DmSNAP190 located between amino acid residues 410 and 483 cross-linked to phosphate position 3 of the U6 PSEA.

Photo-cross-linking of the same six DmSNAPc constructs to position 5 of the U6 PSEA is shown in lanes 25–30 of Figure 2B. DmSNAP190 C-terminal fragment cross-linking is evident in lanes 25–27, and N-terminal fragment cross-linking is clearly seen in lanes 29 and 30. As previously observed (23,25), however, the band in lane 28 is ambiguous because the N- and C-terminal fragments of construct C migrate to the same position on the gel (lanes 5 and 14). We therefore performed an experiment like ones described in earlier work to resolve this ambiguity (23,25). We carried out photo-cross-linking with DmSNAPc that contained one of three different DmSNAP190 constructs: either untagged DmSNAP190, N-terminally tagged DmSNAP190 or C-terminally tagged DmSNAP190, each with the hydroxylamine cleavage site at position 358 (shown at the top of Figure 2C). The tags specifically increased the lengths of the N-terminally tagged fragment and the C-terminally tagged fragment by ∼2.3 and ∼5.7 kDa respectively relative to the untagged fragments. Photo-cross-linking reactions to phosphate position 5 with these three constructs were then run side-by-side on the same gel (Figure 2C, lanes 4–6). The cross-linked radiolabeled fragment ran more slowly when the construct was N-terminally tagged than when the DmSNAP190 construct was untagged or C-terminally tagged. This result allowed us to conclude that, when protein cleavage was at residue 358, the N-terminal fragment of DmSNAP190 cross-linked to phosphate position 5. The pattern of the converse result (e.g. if cross-linking had occurred to the C-terminal fragment) is represented in Figure 2C, lanes 1–3, which exemplifies the C-terminal cross-linking pattern expected for phosphate position 3. Taken together, the results indicated that amino acids of DmSNAP190 between residues 306 and 358 cross-linked to phosphate position 5 of the U6 PSEA.

Results of experiments with the cross-linker at phosphate position 7 of the U6 PSEA are shown in Figure 2B, lanes 31–36. Only the C-terminal fragment cross-linked when DmSNAP190 was cleaved following either residue 168 or 247 (lanes 31 and 32). However, when cleavage was at residue 305 (construct B), both the N-terminal and C-terminal fragments were found to cross-link, although the N-terminal fragment cross-linked more intensely (Figure 2B, lane 33). This means that phosphate position 7 of the U6 PSEA was able to cross-link to amino acid residues of DmSNAP190 both N-terminal and C-terminal of residue 305. When cleavage occurred at positions 409 or 483 (lanes 35 and 36), only the N-terminal fragments cross-linked to phosphate position 7.

Due to the ambiguity associated with cleavage of the protein at position 358, the data shown in Figure 2B, lanes 31–36, could not formally exclude the possibility that phosphate position 7 might cross-link between residues 358 and 409 as well as N-terminal of position 305. However, the results shown in Figure 2C, lanes 7–9, indicate that cross-linking of phosphate position 7 occurred only to the N-terminal side of position 358.

By taking all of these data into account, we conclude that phosphate position 7 of the U6 PSEA cross-linked strongly to residues of DmSNAP190 localized between 248 and 305 and cross-linked with less intensity to residues between 306 and 358. These results, together with those from phosphate positions 3 and 5, are summarized in Figure 2D and are in fact identical to those obtained in earlier work with phosphate positions 3, 5 and 7 of the U1 PSEA (25). Therefore, the same domains of DmSNAP190 are in close proximity to phosphate positions 3, 5 and 7 when DmSNAPc binds to either the U1 or U6 PSEA.

### Identification of DmSNAP190 Myb domain repeats that cross-link to U6 PSEA positions 8, 10 and 12 of the template DNA strand

**Figure 3.**
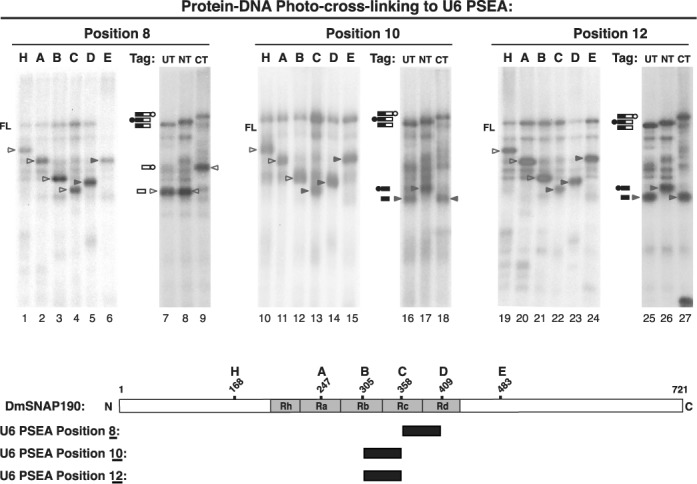
Mapping DmSNAP190 Myb repeat domains that cross-link to U6 PSEA positions 8, 10 and 12 of the template strand. Lanes 1–9, lanes 10–18 and lanes 19–24 show autoradiography results of photo-cross-linking DmSNAP190 NG variants to U6 PSEA phosphate positions 8, 10 and 12, respectively. Lanes and bands are labeled as indicated in the legend of Figure 2. Unshaded arrowheads point at bands corresponding to cross-linked C-terminal fragments, and shaded arrowheads point out cross-linked N-terminal fragments. The diagram at the bottom of the figure indicates the region of DmSNAP190 that cross-linked to each of positions 8, 10 and 12 of the U6 PSEA.

We next investigated the cross-linking of DmSNAP190 to phosphate position 8 of the template strand of the U6 DNA. The pattern of cross-linking (Figure 3, lanes 1–9) revealed that position 8 cross-linked C-terminal to residue 306 (construct B, lane 3) but N-terminal to residue 409 (construct D, lane 5). Data shown in lanes 7–9 clarified that the cross-linking was localized to a region of DmSNAP190 C-terminal of residue 358. We therefore conclude that U6 PSEA position 8 cross-linked to amino acid residues of DmSNAP190 localized between positions 359 and 409.

We next examined the cross-linking pattern of phosphate position 10 of the U6 PSEA to DmSNAP190. The results shown in Figure 3 lanes 10–15 were remarkably similar to those seen for position 8 in lanes 1–6. However, the results in lanes 16–18 were the converse of those observed in lanes 7–9. That is, position 10 cross-linked to the N-terminal fragment when protein cleavage was at position 358. Thus, phosphate position 10 cross-linked to amino acid residues of DmSNAP190 located between positions 306 and 358.

The cross-linking pattern of U6 PSEA phosphate position 12 to DmSNAP190 is shown in Figure 3 lanes 19–27. For each of the DmSNAP190 constructs, the results were identical to those obtained with position 10 as described above. Thus, position 12 of the U6 PSEA, like position 10, cross-linked to the region of DmSNAP190 located between amino acid residues 306 and 358. The cross-linking results for U6 PSEA positions 8, 10 and 12 are summarized in the schematic diagram at the bottom of Figure 3. Notably, these are the same regions of DmSNAP190 that cross-linked to phosphate positions 8, 10 and 12 of the U1 PSEA (23,25). Together with the results discussed in the preceding section, this suggests that phosphate positions 3, 5, 7, 8, 10 and 12 contact similar domains of DmSNAP190 on both the U1 and U6 PSEAs.

### Identification of DmSNAP190 domains that cross-link to U6 PSEA positions 2 and 4 of the template DNA strand

**Figure 4.**
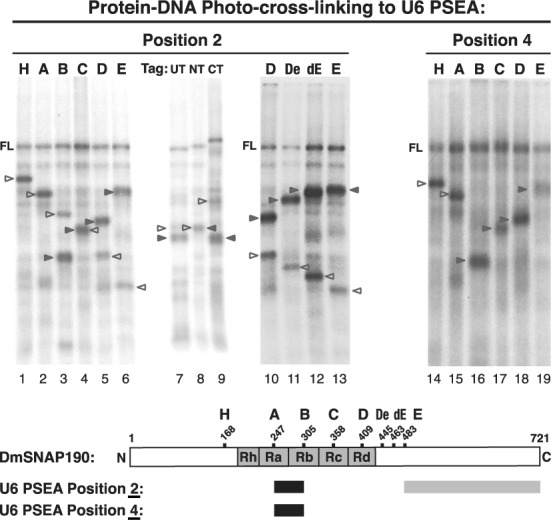
Mapping DmSNAP190 domains that cross-link to U6 PSEA positions 2 and 4 of the template strand. Lanes 1–13 and lanes 14–19 show autoradiograms of the results of photo-cross-linking DmSNAP190 NG variants to U6 PSEA phosphate positions 2 and 4, respectively. Lanes and bands are labeled as indicated in the legend of Figure 2. In lanes 3–13, both the N- and C-terminal fragments cross-linked to position 2. The diagram at the bottom of the figure indicates the region of DmSNAP190 that cross-linked to positions 2 and 4 of the U6 PSEA. Dark shading, stronger cross-linking; lighter shading, weaker cross-linking.

When DmSNAPc binds to a U6 PSEA, DmSNAP190 can be cross-linked to phosphate positions 2 and 4; those cross-links do not occur when DmSNAPc binds to a U1 PSEA (14). Experiments to localize domains of DmSNAP190 that cross-link to these positions in a U6 PSEA are shown in Figure 4. The cross-linking pattern shown in lanes 1- 6 revealed that cross-linking occurred C-terminal of amino acid residue 247 (construct A, lane 2). When DmSNAP190 was cleaved at position 305 (construct B, lane 3), the strongest cross-linking was to the N-terminal fragment. Consistent with this, strong bands corresponding to N-terminal fragments were also observed in lanes 4–6, indicating that strong cross-linking occurred between residues 248 and 305.

Interestingly, a less intense band consistent with cross-linking to the fragment C-terminal of residue 305 was also observed in lane 3. Although a C-terminal cross-linked fragment would be obscured in lane 4, bands consistent with C-terminal fragment cross-linking were observed in lanes 5 and 6 (clear arrowheads). These results suggested that the domain of DmSNAP190 C-terminal of residue 483 was cross-linking to position 2 of the U6 PSEA. Moreover, the data in lanes 7–9 of Figure 4 were consistent with the possibility that the N-terminal and C-terminal fragments both cross-linked to position 2 when the protein was cleaved at residue 358. To further investigate the authenticity of the C-terminal bands observed in lanes 1–9, we employed two new DmSNAP190 constructs (De and dE) that could be cleaved by hydroxylamine following residues 445 or 463. Figure 4 (lanes 10–13) shows results obtained with these constructs run alongside constructs D and E. In each case, both the C-terminal fragment as well as the N-terminal fragment were observed to cross-link. These results provide convincing evidence that phosphate position 2 of the U6 PSEA cross-linked not only to a domain of DmSNAPc between residues 248 and 305 but cross-linked also to a domain of DmSNAP190 C-terminal of residue 483.

The pattern of cross-linking of DmSNAP190 to position 4 is shown in Figure 4 lanes 14–19. The pattern is similar to position 2 except there is no evidence of cross-linking to any region C-terminal of residue 305. A summary of the cross-linking results for U6 PSEA phosphate positions 2 and 4 is shown at the bottom of Figure 4. Both of these positions cross-linked to a domain of DmSNAP190 between amino acid residues 248 and 305. In addition, the C-terminal domain of DmSNAP190 (bounded by residues 484–721) was capable of cross-linking to position 2.

### Identification of DmSNAP190 Myb domain repeats that cross-link to U6 PSEA positions 11 and 13 of the non-template DNA strand

**Figure 5.**
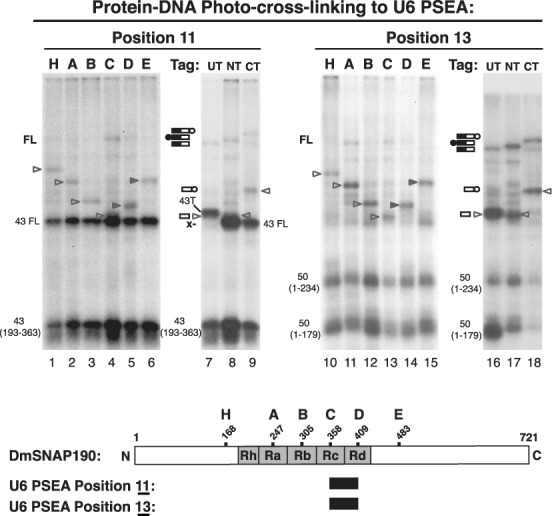
Mapping DmSNAP190 Myb repeats domains that cross-link to U6 PSEA positions 11 and 13 of the non-template strand. Lanes 1–9 and lanes 10–18 show autoradiograms of the results of photo-cross-linking DmSNAP190 NG variants to U6 PSEA phosphate positions 11 and 13, respectively. Lanes and bands are labeled as indicated in the legend of Figure 2. Unshaded arrowheads point out the bands corresponding to cross-linked C-terminal fragments and shaded arrowheads point to the bands corresponding to N-terminal fragments. Positions 11 and 13 cross-link not only to DmSNAP190 but also DmSNAP50 and DmSNAP43, respectively (14,23,24). 43FL indicates the band that arises from cross-linking to full-length untagged DmSNAP43. In lane 7, 43T points out a band that arises from cross-linking to N-terminally tagged full-length DmSNAP43 that was incorporated into the DmSNAPc used in this lane as a means of purification when DmSNAP190 was not tagged. The x alongside lane 7 indicates the absence of a band that would be expected if cross-linking had occurred to the N-terminal fragment of construct UT. The designations 43(193–363), 50(1–234) and 50(1–179) indicate the positions of cross-linked DmSNAP43 and DmSNAP50 hydroxylamine digestion products with the numbers in parentheses indicating the amino acid positions at the beginning and end of those fragments. The diagram at the lower part of the figure indicates the region of DmSNAP190 that cross-linked to positions 11 and 13 of the U6 PSEA.

When DmSNAPc binds to DNA, DmSNAP190 cross-links to phosphate positions 11 and 13 of the U6 PSEA but not to those same positions of a U1 PSEA (Figure 1B) (14). To map out the domains of DmSNAP190 that cross-link to positions 11 and 13 of the U6 PSEA, the experiments shown in Figure 5 were carried out. Because positions 11 and 13 cross-linked not only to DmSNAP190 but also to DmSNAP43 and DmSNAP50 respectively (14), additional bands corresponding to those two smaller subunits appear on the gel. From previous work and knowledge of the primary structure of those subunits (23,25), it is possible to identify the DmSNAP43 and DmSNAP50 bands on the gel and, except when overlapping bands occur, avoid confusion with the DmSNAP190 data.

When cross-linking was carried out with phosphate position 11 of the U6 PSEA (Figure 5, lanes 1–9), the strongest bands corresponded to cross-links with full length (undigested) DmSNAP43 and to the DmSNAP43 fragment encompassed by residues 193–363 in accord with previous observations (23). Nevertheless, bands could also be observed that corresponded to the fragments derived from hydroxylamine digestion of the DmSNAP190 constructs (designated by arrowheads). By the same reasoning previously applied, the results shown in lanes 1–6 of Figure 5 localized the cross-linking of position 11 to the region of DmSNAP190 bounded by residues 306 and 409.

To ascertain whether this cross-linking was to the region of the protein N-terminal or C-terminal of residue 358, the cross-linking experiments shown in lanes 7–9 were performed. Although some of the DmSNAP190 bands were partially obscured by the much stronger DmSNAP43 bands, the evidence was consistent with cross-linking that occurred C-terminal of position 358. First, and most importantly, in lane 9 there was a band that corresponded to the tagged C-terminal fragment. Second, there were faint ‘shoulder’ bands where the untagged C-terminal fragment of DmSNAP190 was expected to run just below the tagged DmSNAP43 band in lane 7 and just above the untagged DmSNAP43 band in lane 8. Finally, and significantly, there was no band in lane 7 that would correspond to the mobility of an untagged N-terminal DmSNAP190 fragment (that would run at the position denoted by ‘x’). Therefore, we conclude that phosphate position 11 of the U6 PSEA cross-linked to the domain of DmSNAP190 that contains amino acid residues 359–409.

Experiments in which the cross-linker was placed at position 13 in the U6 PSEA are shown in Figure 5 lanes 10–18. Despite the presence of DmSNAP50-derived bands in the lower part of the gel, bands corresponding to DmSNAP190 fragments were easily discerned in the upper part of the gel. In lanes 1-10, the DmSNAP190 cross-linking pattern was exactly the same as in lanes 1–9, respectively. We therefore conclude that both phosphate positions 11 and 13 of the U6 PSEA cross-linked to the domain of DmSNAP190 bounded by amino acid residues 359–409. These results are summarized schematically at the bottom of Figure 5.

### Identification of DmSNAP190 domains that cross-link to positions 17, 19, and 25 of the non-template DNA strand and to positions 14, 22, and 24 of the template DNA strand of the U6 PSEA

**Figure 6.**
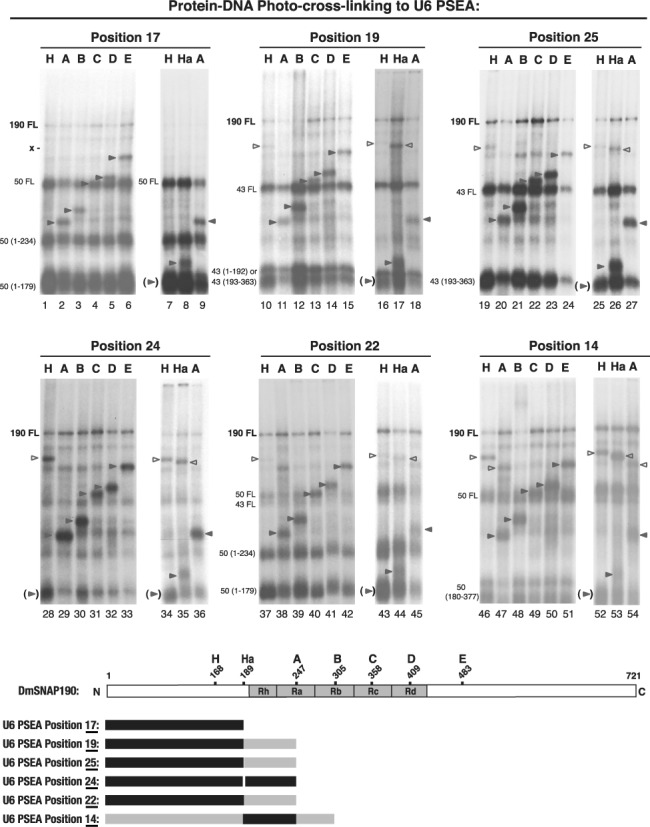
Localization of DmSNAP190 domains that cross-link to positions 17, 19 and 25 of the non-template strand and to positions 14, 22 and 24 of the template strand of the U6 PSEA. Autoradiograms are shown representing the results of photo-cross-linking various DmSNAP190 constructs to U6 PSEA phosphate positions 17, 19, 25, 24, 22 and 14. Lanes and bands are labeled as indicated in the legend of Figure 2. Unshaded arrowheads point out the bands corresponding to cross-linked C-terminal fragments and shaded arrowheads points to the cross-linked N-terminal fragments. 190FL indicates the position of the full-length undigested N-tagged DmSNAP190. The labels 43FL and 50FL point out bands that correspond to cross-linked full-length DmSNAP43 and DmSNAP50, respectively. The numbers in parentheses following 50 or 43 [e.g. 50(1–234)] indicate the positions of hydroxylamine digestion fragments of DmSNAP43 or DmSNAP50 bounded by those amino acid residues. The x alongside lane 1 indicates the absence of a band that would be expected if cross-linking had occurred to the C-terminal fragment of construct H. The shaded arrowheads in parentheses point to the location of a surmised DmSNAP190 N-terminal fragment derived from construct H (in lanes 7, 16, 25, 34, 43 and 52) that would be cross-linked but is obscured by the strong bands arising from digestion products of DmSNAP50 or DmSNAP43. The diagram at the bottom of the figure indicates the region of DmSNAP190 that cross-linked to each of positions 17, 19, 25, 24, 22 and 14 of the U6 PSEA. Dark shading, stronger cross-linking; lighter shading, weaker cross-linking.

Cross-linking data for the remaining six phosphate positions of the U6 PSEA that cross-link to DmSNAP190 are shown in Figure 6.

*Position 17.* At position 17, DmSNAP50 cross-links much more intensely than DmSNAP190 (14). Nevertheless, the bands derived from the DmSNAP190 constructs can be discerned in most lanes and can be surmised when occluded by DmSNAP50 bands. Figure 6 lanes 2–6 established that phosphate position 17 of the U6 PSEA cross-linked to the N-terminal side of residue 247 (construct A). The lack of a high molecular weight band in lane 1 at the position marked by an ‘x’ furthermore suggests that phosphate position 17 cross-linked N-terminal of amino acid residue 168. However, the N-terminal fragment derived from construct H would be occluded near the bottom of the gel by the very strong band derived from DmSNAP50. To shed further light on the region of DmSNAP190 that cross-linked to position 17, an experiment was carried out with the construct Ha (lanes 7–9). In this case, the N-terminal fragment of Ha resolved from the DmSNAP50 bands and revealed that phosphate 17 cross-linked, after hydroxylamine digestion of the protein, to the N-terminal fragment of DmSNAP190 localized between residues 1 and 189 (and most likely between 1 and 168). No cross-linking C-terminal of position 189 was observed.

*Position 19.* The cross-linking pattern of U6 PSEA phosphate position 19 to DmSNAPc is shown in Figure 6 lanes 10–18. Besides the bands derived from the DmSNAP190 constructs, several bands corresponding to full-length and digested fragments of DmSNAP43 were observed, but these had only a minimal effect on the DmSNAP190 analysis. The DmSNAP190 fragments formed a pattern revealing that position 19 of the U6 PSEA cross-linked to the N-terminal side of the cut site in construct A at residue 247 (lane 11). In lane 10, a very weak high molecular weight band could be observed that corresponded to a cross-link to the C-terminal fragment of construct H. This band indicated that weak cross-linking occurred C-terminal to the hydroxylamine cut site at residue 168. However, it still remained possible that there could also be cross-linking occurring N-terminal of residue 168. To investigate this possibility, the experiments shown in Figure 6 lanes 16–18 were performed. In this case, stronger cross-linking to the N-terminal fragment and weaker cross-linking to the C-terminal fragment were observed when the hydroxylamine cut site was at position 189 (lane 17). From these results, we conclude that position 19 of the U6 PSEA cross-linked most intensely to the region of DmSNAP190 located between amino acid residues 1 and 189 and with less intensity to the domain of DmSNAP190 between residues 190 and 247.

*Position 25.* When the cross-linking reagent was placed at position 25 of the U6 PSEA, the pattern of cross-linking was essentially identical to that obtained with position 19 (compare the DmSNAP190 bands in lanes 19–27 with those in lanes 10–18). Thus, we likewise conclude that phosphate position 25 cross-linked more strongly to the region of DmSNAP190 encompassed by amino acid residues 1–189 and more weakly to the DmSNAP190 domain between residues 190 and 247.

*Position 24.* Placement of the cross-linker at position 24 of the U6 PSEA produced a relatively clear cross-linking pattern (Figure 6, lanes 28–36) because the two smaller subunits of DmSNAPc did not cross-link at this position (14). Cross-linking at position 24 occurred to the N-terminal fragment of constructs A through E (lanes 29–33). A high molecular weight band was clearly observed that corresponded to the cross-linked C-terminal fragment of construct H (lane 28), but the band near the bottom of the gel was darker in lane 28 compared to the non-specific band of similar mobility in the remainder of the lanes, suggesting that cross-linking might also have occurred to the N-terminal fragment derived from construct H. To further assess whether the N-terminal domain of DmSNAP190 cross-linked to position 24, the reactions shown in Figure 6, lanes 34–36, that included the Ha construct were carried out. Lane 35 shows clear evidence for the cross-linking of position 24 to the N-terminal fragment of the Ha construct as well as to the C-terminal fragment. From these results, we conclude that position 24 of the U6 PSEA cross-linked with approximately equal intensities to the N-terminal region of DmSNAP190 between amino acid residues 1 to 189 and to the adjacent region of DmSNAP190 located between residues 190 and 247.

*Position 22.* Results with the cross-linking reagent at phosphate position 22 of the U6 PSEA were similar to those obtained at position 24, except that the cross-linking intensity C-terminal of residue 189 was very, very weak (lanes 43 and 44). Thus, position 22 of the U6 PSEA cross-linked to the N-terminal domain of DmSNAP190 (residues 1–189) and very weakly to the domain of DmSNAP190 encompassed by residues 190 to 247.

*Position 14.* Cross-linker placed at position 14 of the U6 PSEA resulted in perhaps the most complicated pattern observed. The results indicated that when DmSNAP190 was cleaved at sites B, C, D or E, cross-linking was exclusively to the N-terminal fragment, placing the region of DmSNAP190 cross-linking entirely N-terminal of amino acid residue 305 (Figure 6, lanes 48–51). When the hydroxylamine cleavage site was at residue 247, both the N-terminal fragment (more strongly) and the C-terminal fragment (more weakly) cross-linked (lane 47). This indicated that U6 PSEA position 14 cross-linked primarily N-terminal of residue 247, but also cross-linked to the domain of DmSNAP190 located between amino acid residues 248 and 305.

As expected with construct H, cross-linking to the C-terminal fragment was observed (lane 46), but there remained the possibility that cross-linking also occurred to the N-terminal fragment (which would be occluded by the DmSNAP50 fragment near the bottom of the gel). The cross-linking reactions shown in Figure 6 lanes 52–54 shed further light on that situation. When DmSNAPc was cleaved at position 189 (construct Ha, lane 53), stronger cross-linking was observed to the C-terminal fragment, but cross-linking was also observed to the N-terminal fragment. Taking all of the data into consideration, the most consistent interpretation is that position 14 of the U6 PSEA cross-linked most strongly to the domain of DmSNAP190 located between amino acid residues 190 and 247, but that cross-linking also occurred to DmSNAP190 between residues 1 to 189 as well as to residues between 248 and 305. A schematic summary of the results for U6 PSEA positions 17, 19, 25, 24, 22 and 14 is shown at the bottom of Figure 6.

### Comparison of the pattern of DmSNAP190 cross-linking to U1 and U6 PSEAs

**Figure 7.**
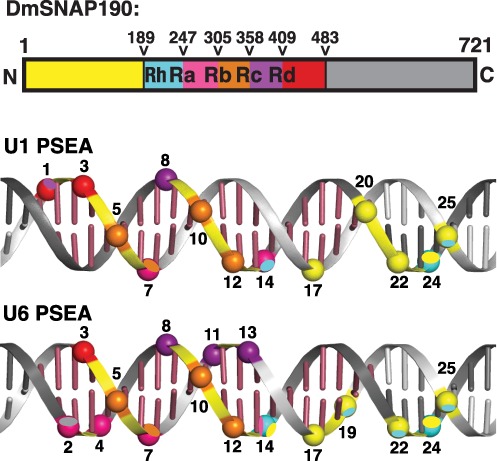
Summary of the mapped regions of DmSNAP190 that contact specific nucleotide positions of the U1 or U6 PSEAs. The DmSNAP190 cross-linking data for the U1 and U6 PSEAs summarized in Supplementary Figure S1 were projected on B-form DNA. Each phosphate position that cross-linked is color-coded to match the color of the domain of DmSNAP190 (as indicated in the schematic at the top of the figure) that cross-linked most strongly to that particular phosphate position. Oval overlays on certain spheres are color-coded to match the color of protein regions that cross-linked with weaker intensity. Raspberry-colored bases represent the 21 bp of the U1 or U6 PSEAs.

Earlier studies identified 16 phosphate positions that cross-linked to DmSNAP190 when DmSNAPc was bound to a U6 PSEA (Figure 1). The results described in Figures 2–6 localized and identified domains of DmSNAP190 that cross-linked to each of these 16 phosphate positions within and downstream of the U6 PSEA. Recently, we carried out a similar study on all 13 positions of the U1 PSEA that cross-linked to DmSNAP190 (23–25). Supplementary Figure S1 is a summary that schematically illustrates the DmSNAP190 domains that cross-link to specific phosphate positions in the U1 or U6 PSEAs. Figure 7 shows these data projected upon B-form DNA double helices that contain either a U1 or a U6 PSEA. The protein domains and the phosphates on the DNA are color-coded to indicate the mapped region of the DmSNAP190 protein that cross-linked to the similarly-colored phosphate spheres on the DNA. The color-coding in this figure corresponds to regions of the protein that are bounded by the hydroxylamine cutting sites and thus extend from the middle of one Myb repeat to the middle of the adjacent Myb repeat. In cases where more than one domain of the protein cross-linked to the DNA, an oval of the color of the more weakly cross-linking domain was overlaid over a portion of the phosphate backbone sphere that was initially colored to match the protein region that most strongly cross-linked.

The location of DmSNAP190 and its domains on the U1 and U6 promoters is similar but there are also significant differences. In both cases, the Rh Myb half-repeat and the DmSNAP190 N-terminal domain (shown in yellow) interacted with the 3′ half of the PSEA (Figure 7). The 3′ half of the PSEA is also the portion of the PSEA that is contacted by the DmSNAP50 and DmSNAP43 subunits (14,23). The 3′ half of the fly PSEA is less conserved than the 5′ half, and it differs at specific nucleotide positions that are ‘conserved to be different’ between the snRNA genes that are transcribed by Pol II and those transcribed by Pol III (20).

Perhaps the most striking difference between the U1 and U6 binding patterns is that unique minor groove-spanning contacts occur on the U6 PSEA that are absent when DmSNAPc binds to the U1 PSEA (Figure 7). On a U6 PSEA, a domain of DmSNAP190 (in purple, amino acid residues 359–409) spans the minor groove between phosphate 8 on the template strand and phosphates 11 and 13 on the non-template strand; this suggests that there is a shift of this domain in the downstream direction (toward the transcription start site) compared to its position on a U1 PSEA. Similarly, a second domain of DmSNAP190 (magenta, residues 248–305) spans the U6 PSEA minor groove between phosphate 7 of the template strand and phosphates 2 and 4 on the non-template strand, suggesting a movement of the corresponding DmSNAP190 domain in the upstream direction compared to its location on the U1 PSEA.

### Model of the differential interaction of DmSNAP190 with the U1 and U6 PSEAs

**Figure 8.**
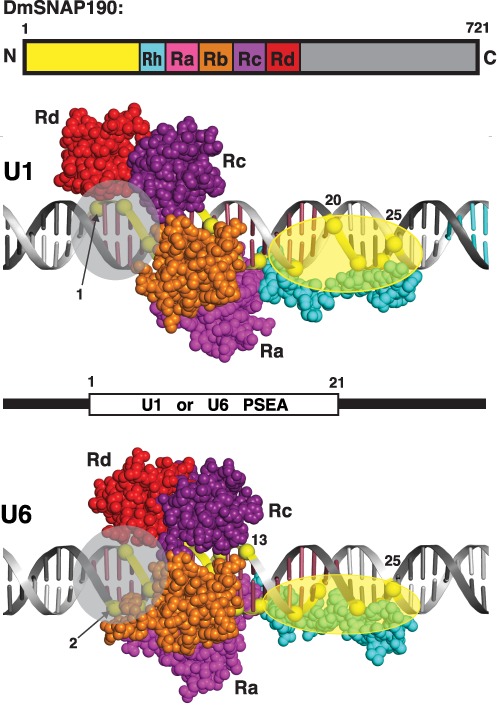
Differential interaction of DmSNAP190 with the U1 and U6 PSEAs. The bar at the top is a schematic diagram of DmSNAP190. Below, models are shown of DmSNAP190 (as a subunit of DmSNAPc) differentially bound to the U1 and U6 PSEAs. Between the models, the location of the 21 bp PSEA within the DNA duplexes is represented. Certain phosphate positions are explicitly labeled; for the other phosphate positions see Figure 1B. Throughout the figure, the N-terminal domain, Rh, Ra, Rb, Rc, Rd and the C-terminal domain of DmSNAP190 are shown in yellow, cyan, magenta, orange, deep purple, red and gray colors, respectively.

There are no molecular structures available for the components of DmSNAPc or of its orthologs from other species. However, there is a co-crystal structure available for the DNA-binding domain of the c-Myb protein bound to DNA (Protein Data Bank entry 1H88) (28). Each Myb repeat consists of three alpha helices separated by turns, and each Myb repeat is separated from adjacent Myb repeats by a flexible linker. We previously generated a three-dimensional theoretical model of the DmSNAP190 Myb repeats bound to a U1 PSEA (25) by using a web-based structural alignment tool (SWISS-MODEL repository) (31) to thread the DmSNAP190 Myb repeat sequences onto the structure of the Myb repeats of the c-Myb protein. We have now performed the same type of structure-building to generate a model of DmSNAP190 on the U6 PSEA (Supplementary Figure S2 and Figure 8). We have also revised the original U1 model to bring it better into accord with the constraints of the U6 model (Figure 8). All protein data files were visualized and manipulated using PyMOL (Schroedinger LLC, New York, NY).

Supplementary Figure S2 shows a model of DmSNAP190 bound to the U6 PSEA that follows the color scheme of Figure 7 and of Supplementary Figure S1, which is based upon the hydroxylamine fragmentation pattern of DmSNAP190. The model was developed by manually placing the Myb repeat structures (modeled using the SWISS-MODEL repository software) into the proximity of the cross-linked phosphates in accordance with the color code of Figure 7. To alternatively depict the model in a manner more easily understood with respect to the individual Myb repeats, we then re-colored the protein model so that the boundaries of the colors would correspond to the boundaries of the individual Myb repeats. The lower part of Figure 8 shows the exact same U6 model as in Supplementary Figure S2 but re-colored to depict each of the individual Myb repeats in its own distinct color. The upper part of Figure 8 shows DmSNAP190 modeled in the same way but bound to the U1 PSEA.

While developing both the U1 and U6 models, we retained intact the predicted structures of each of the complete Myb repeats Ra, Rb, Rc and Rd; only the flexible linkers that connect the Myb repeats (32) were manipulated to generate models that would be consistent with the U1 and U6 cross-linking data. We took a liberty only with the Rh half-repeat that contains just the last two of the three conserved helices present in a full Myb repeat. To permit the Rh region of the protein to stretch over the relatively long length of DNA to which this half-repeat was able to cross-link on both the U1 and U6 PSEAs, it was necessary to ‘straighten out’ the turn that links the two helices. This is reasonable because the stability of the overall structure of an individual Myb repeat is dependent upon a hydrophobic core whose formation depends upon contributions from all three helices within the repeat (32).

Work from other investigators demonstrated that the R2 and R3 Myb repeats of c-Myb interact with DNA by inserting a recognition alpha helix into the major groove of the DNA (28–29,32). We have followed that scheme precisely for the Rb repeat of DmSNAP190 and placed its ‘recognition helix’ into the major groove at the same position in both the U1 PSEA and in the U6 PSEA. This placed the recognition helix of Rb in proximity to base pairs 5, 6, 7 and 8 of the U1 and U6 PSEAs. Interestingly, these are the base pairs that are the most stringently conserved among all the PSEAs of both the Pol II- and Pol III-transcribed snRNA genes of *D.* *melanogaster*. In fact, these are also the most conserved bases of all the other insect species’ PSEAs that have been analyzed (20,33). It is further worth noting that the cross-linking patterns most similar between the U1 and U6 PSEAs are the cross-links between the DNA and the Rb repeat, as opposed to the other domains of DmSNAP190 (Figure 7). Thus, interactions between the Rb recognition helix and the base pairs in the vicinity of PSEA positions 5 through 8 likely represent the most conserved core of DmSNAP190 interactions utilized by both the U1 and U6 PSEAs.

On a U1 PSEA (upper part of Figure 8), repeats Ra, Rb, Rc and Rd follow the contour of the major groove of the DNA between approximately positions 1 through 12 of the U1 PSEA. In contrast, on a U6 PSEA, repeat Rc seems to move slightly in the downstream direction (toward the transcription start site) whereas repeat Ra moves slightly in the upstream direction. As a result of this movement, Rc spans the minor groove of the U6 PSEA from phosphate 8 to phosphates 11 and 13. Likewise, specific regions of repeats Ra and Rb (primarily the unusually long flexible linker that joins these two repeats) span the minor groove from phosphates 5 and 7 to contact phosphates 2 and 4. Within the constraints of the conformation of the Myb repeats and molecular bond distances, it is not feasible to place the Rc or Ra ‘recognition helices’ deep into the major groove and at the same time have these repeats span the minor groove as demanded by the U6 cross-linking data. In fact, the R1 repeat of c-Myb in the nuclear magnetic resonance and co-crystal structures of c-Myb does not directly interact with the major groove of the DNA (28,32), so there is precedent for the lack of direct interaction between the major groove of the DNA and the Myb repeat third helix.

We have considered the possibility that the DNA of the U1 and U6 PSEAs could be differentially bent, which in principle could partially account for the unique Myb repeat minor groove-spanning interactions observed for U6. However, extensive experiments to examine the bending of the U1 and U6 promoter DNA while bound to DmSNAPc indicated that there was only a minor bend in the DNA, and the magnitude and direction of the bend were very similar for DmSNAPc-bound U1 and U6 promoter DNA (34). Furthermore, base positions 1 through 12 in the PSEAs of both the Pol II- and Pol III-transcribed snRNA genes of *D. melanogaster* are very strongly conserved in nucleotide sequence (9,20). Thus, it would be very surprising if the nearly identical sequences in the 5′ half of the PSEA (i.e. the region contacted by the Ra, Rb, Rc and Rd repeats) would be differentially bent in the U1 and U6 promoters. Although it is unlikely that the DNA bound by DmSNAPc exists as simple B-form DNA over its entire length as represented in Figure 8, in the absence of any additional information it seems reasonable to utilize B-form DNA for the molecular modeling.

Another interesting difference in the U1 and U6 pattern of contacts is that the N-terminal domain of DmSNAP190 (residues 1–189, shown in yellow in Figure 8) cross-linked to the phosphate at position 20 when DmSNAPc was bound to a U1 PSEA but did not cross-link with this same position of the U6 PSEA. This N-terminal domain of DmSNAP190 made instead a contact to position 19 in the U6 PSEA but did not contact position 19 in the U1 PSEA. Because phosphate 19 is on the non-template strand but phosphate 20 is on the template strand, these two phosphate positions exist on nearly opposite faces of the DNA double helix (see Figure 1B). This suggests that the DmSNAP190 N-terminal domain, when viewed from upstream of the PSEA looking toward the transcription start site, was rotated slightly in the clockwise direction around the DNA on a U6 PSEA relative to its location on a U1 PSEA. In further accord with such a movement, the Rh repeat of DmSNAP190 was also found to make an additional new contact with position 19 of the U6 PSEA that did not occur in the case of U1. This suggests that Rh, in concert with the adjacent N-terminal domain, also made a similar clockwise rotation on the U6 DNA compared to the U1 DNA.

A surprising result from our U6 PSEA cross-linking data was that the domain of DmSNAP190 C-terminal to the Myb repeats cross-linked to phosphate position 2 of the U6 PSEA. No cross-linking of this C-terminal domain (represented in gray in Figure 8) was observed to DNA of the U1 PSEA. However, we cannot rule out the possibility that this domain of DmSNAP190 might cross-link to phosphate positions in the U1 PSEA that were not examined.

## DISCUSSION

### Model of the differential interaction of the DmSNAP complex with the U1 and U6 PSEAs

**Figure 9.**
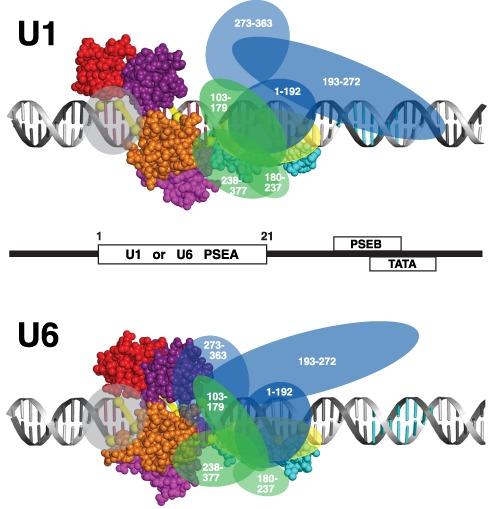
A model of the complete DmSNAP complex differentially bound to U1 and U6 promoter sequences. DmSNAP190 is represented as shown in Figure 8. The green and blue ovals represent domains of DmSNAP50 and DmSNAP43 respectively. The numbers inside the ovals indicate the amino acid positions at the beginning and at the end of DmSNAP50 and DmSNAP43 domains previously mapped on the U1 and U6 PSEAs by site-specific protein–DNA photo-cross-linking (9,23). The bases colored cyan represent the locations of the PSEB and TATA sequences in the U1 and U6 DNA respectively. DmSNAPc appears to form a more ‘closed’ structure on the U6 PSEA compared to the U1 PSEA, suggesting that distinct faces of the DmSNAPc subunits may be exposed (or occluded) on U1 and U6 promoters.

In previous work, protein domains within DmSNAP50 and DmSNAP43 that cross-linked to specific nucleotide positions of the U1 and U6 PSEAs were mapped and localized (23). As a result, we determined the specific positions in the DNA where individual domains of these two smaller subunits interact with the U1 and U6 PSEAs. To obtain a better understanding of the binding of the entire DmSNAP complex to U1 and U6 promoter sequences, we superimposed that earlier data for the two smaller subunits upon the results obtained with DmSNAP190. These results are shown in Figure 9. On a U1 PSEA, a domain of DmSNAP43 bounded by amino acid residues 193–272 contacted the DNA far downstream of the U1 PSEA in the region surrounding the PSEB (9,15,23). Those contacts were absent when DmSNAPc was bound to a U6 PSEA. Conversely, a second domain of DmSNAP43 bounded by amino acid residues 273–363 cross-linked to phosphates 11 and 16 on the U6 PSEA but not when bound to a U1 PSEA. In the case of DmSNAP50, there were also significant yet more subtle differences observed in its positioning on the U1 and U6 PSEAs (9,14,23).

Based upon all available site-specific protein–DNA photo-cross-linking data, the illustrations in Figure 9 show comprehensive schematic models of DmSNAPc bound differentially to U1 or U6 PSEAs. On the U1 PSEA, DmSNAPc appears to adopt a more open conformation that allows residues 193–272 of DmSNAP43 to contact the DNA in the region of the PSEB. In contrast, on the U6 PSEA new protein–DNA contacts occur at phosphates 11 and 13 (with the Rc repeat of DmSNAP190) and at phosphates 11 and 16 (with the C-terminal domain of DmSNAP43). As a result DmSNAPc adopts a more ‘closed’ conformation with potentially new interfacial interactions among the subunits, particularly between DmSNAP190 and DmSNAP43. (Note that DmSNAP190 and DmSNAP43 both cross-link to phosphate position 11 on the U6 PSEA, but neither cross-link to position 11 of the U1 PSEA.) As a consequence of DmSNAP190 (Rc domain) and DmSNAP43 (273–363) moving toward each other on the U6 PSEA, the interactions of DmSNAP43 (193–272) with the phosphates that surround the PSEB downstream of the U1 PSEA no longer occur.

### Compatibility of the model with subunit–subunit interactions and with data from the human system

DmSNAPc subunit–subunit interaction studies performed in the absence of DNA previously demonstrated that DmSNAP43 residues 1–172 interact with residues within the Myb repeat domain of DmSNAP190 (9,30). In agreement with that observation, the protein–DNA cross-linking data place the N-terminal domain of DmSNAP43 in close proximity to the Rh Myb repeat on both the U1 and U6 PSEAs (Figure 9). Likewise, previous subunit interaction studies found that residues 110–377 of DmSNAP50 interact with the N-terminal domain (residues 63–176) of DmSNAP190 (9,30). In close accord with that data, the protein–DNA cross-linking studies similarly place this same region of DmSNAP50 in close proximity to the DmSNAP190 N-terminal domain on both the U1 and U6 PSEAs (Figure 9). Finally, the subunit interaction studies also indicated that the N-terminal domain of DmSNAP43 (residues 1–172) interact with residues 110–377 of DmSNAP50 (9,30). The protein–DNA photo-cross-linking studies likewise indicated that these same regions of DmSNAP43 and DmSNAP50 are in close proximity on both the U1 and U6 PSEAs (Figure 9). Thus, the subunit–subunit interaction studies and the photo-cross-linking data are very consistent with each other for all three DmSNAPc subunits.

In accord with the models shown in Figure 9, recent ChIP-seq studies combined with previous subunit–subunit interaction studies in the human system have also provided an indication that SNAP190 is oriented with its N-terminal domain facing toward the transcription start site (35). Finally, additional data from the human system indicated that the N-terminal domain of SNAP190 was involved in recruiting the TATA box binding protein (TBP) to the U6 promoter (36). Our studies place this N-terminal domain of DmSNAP190 at the 3′ end of the U6 PSEA where it would be ideally positioned to interact with TBP.

### Concluding remarks

By combining site-specific protein–DNA photo-cross-linking with site-specific digestion of the three subunits of DmSNAPc, we have obtained data that allow us to formulate a model of how DmSNAPc differentially interacts with U1 and U6 promoter sequences. From these data, we suspect that DmSNAPc is a quite flexible protein complex in solution that, upon binding to DNA, can preferentially adopt one of two distinct conformations depending upon the DNA sequence to which it is bound. Although DmSNAP190 is the only subunit of the three that has an obvious DNA-binding domain, it seems likely that DmSNAP43 and DmSNAP50 (and perhaps the N-terminal domain of DmSNAP190 as well) also bind sequence-specifically to DNA. Indeed, most of the nucleotide differences between the U1 and U6 PSEAs are concentrated in the 3′ half of the PSEAs, which is occupied by all three subunits. We suspect that the DNA sequence differences result in a concerted movement of DmSNAP43 and DmSNAP50 (along with the N-terminal domain and Rh repeat of DmSNAP190) on the DNA. It is our belief that this protein movement at the 3′ half of the PSEA is then transduced along the DmSNAP190 backbone to result in the differential displacement of the Ra-Rd Myb repeats, which can then be reflected in the different DmSNAP190 cross-linking patterns on the 5′ half of the U1 and U6 PSEAs.

Because the DNA sequence of the PSEA plays an important role in determining the RNA polymerase specificity of the U1 and U6 promoters, it seems likely that RNA polymerase specificity is mediated through the different conformations of DmSNAPc on the U1 and U6 promoter sequences. Determining the mechanisms by which the different DmSNAPc conformations recruit different RNA polymerases will be an important focus of future investigation.

## SUPPLEMENTARY DATA

Supplementary Data are available at NAR Online.

SUPPLEMENTARY DATA
