# Supplementary material for: The Myb domain of the largest subunit of SNAPc adopts different architectural configurations on U1 and U6 snRNA gene promoter sequences
Source: Nucleic Acids Res. 2014 Oct 16;42(20):12440–54. doi: 10.1093/nar/gku905 (PMC4227766; doi:10.1093/nar/gku905)
Supplement: SUPPLEMENTARY DATA [file supp_42_20_12440__index.html]

The Myb domain of the largest subunit of SNAPc adopts different architectural configurations on U1 and U6 snRNA gene promoter sequences — The Myb domain of the largest subunit of SNAPc adopts different architectural configurations on U1 and U6 snRNA gene promoter sequences — SUPPLEMENTARY DATA 

# The Myb domain of the largest subunit of SNAPc adopts different architectural configurations on U1 and U6 snRNA gene promoter sequences

## SUPPLEMENTARY DATA

**Files in this Data Supplement:**

- SUPPLEMENTARY DATA
