# Supplementary material for: The Myb domain of the largest subunit of SNAPc adopts different architectural configurations on U1 and U6 snRNA gene promoter sequences
Source: Nucleic Acids Res. 2014 Oct 16;42(20):12440–54. doi: 10.1093/nar/gku905 (PMC4227766; doi:10.1093/nar/gku905)
Supplement: SUPPLEMENTARY DATA [file supp_gku905_nar-02243-a-2014-File011.pdf]

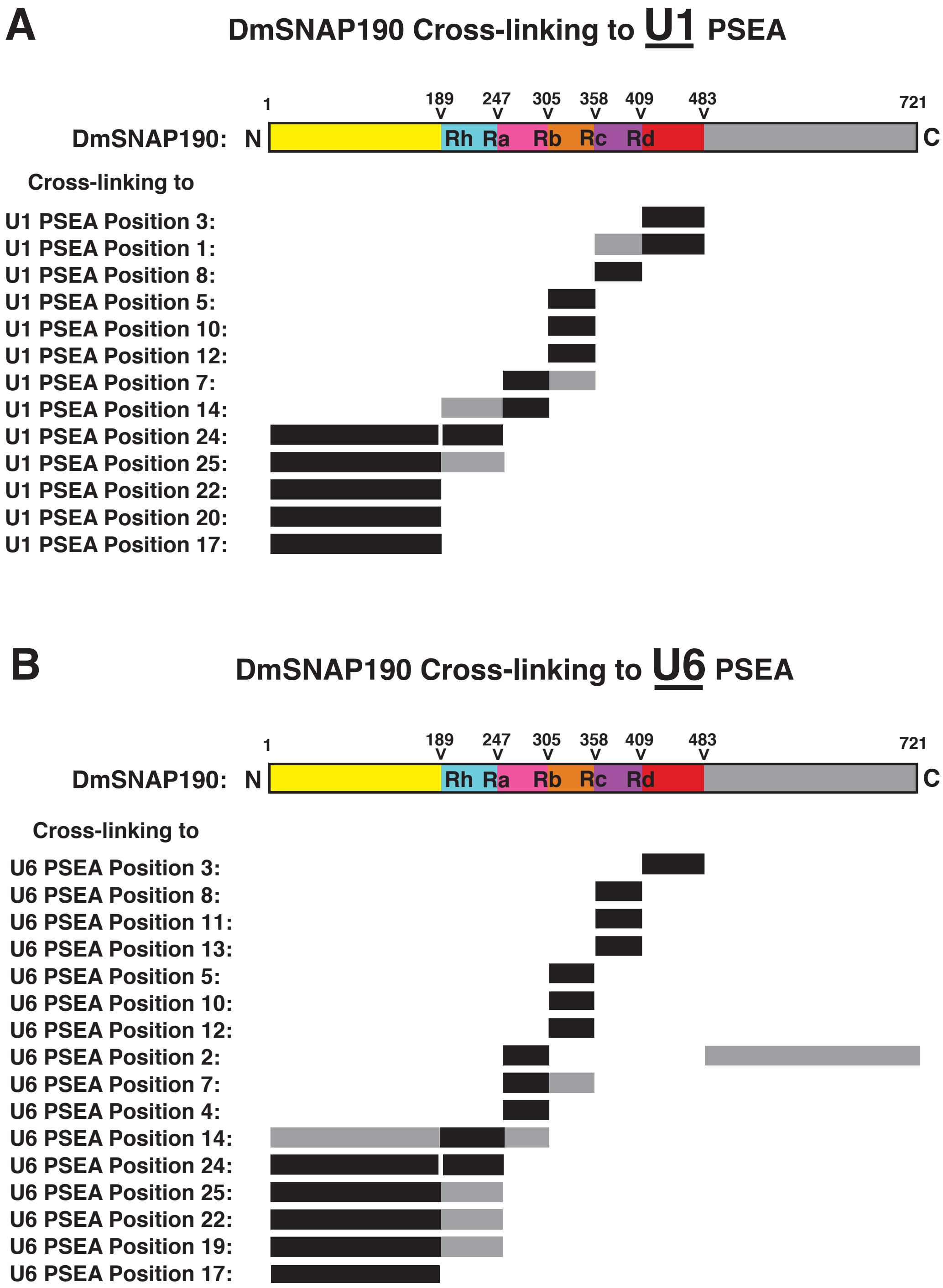

FIGURE S1. DmSNAP190 cross-linking to U1 and U6 PSEAs. (A) and (B) respectively show summaries of the regions of DmSNAP190 that interact with specific nucleotide positions of the U1 and U6 PSEAs as indicated by the notations at the left in each section of the figure. The bars at the top of (A) and (B) represent schematic diagrams of DmSNAP190 with the actual mapped regions depicted in different colors separated by the hydroxylamine cleavage sites. Stronger cross-linking is represented by darker shading, and weaker cross-linking is indicated by lighter shading. The U1 mapping results shown in part (A) are based upon data taken from references 23-25.

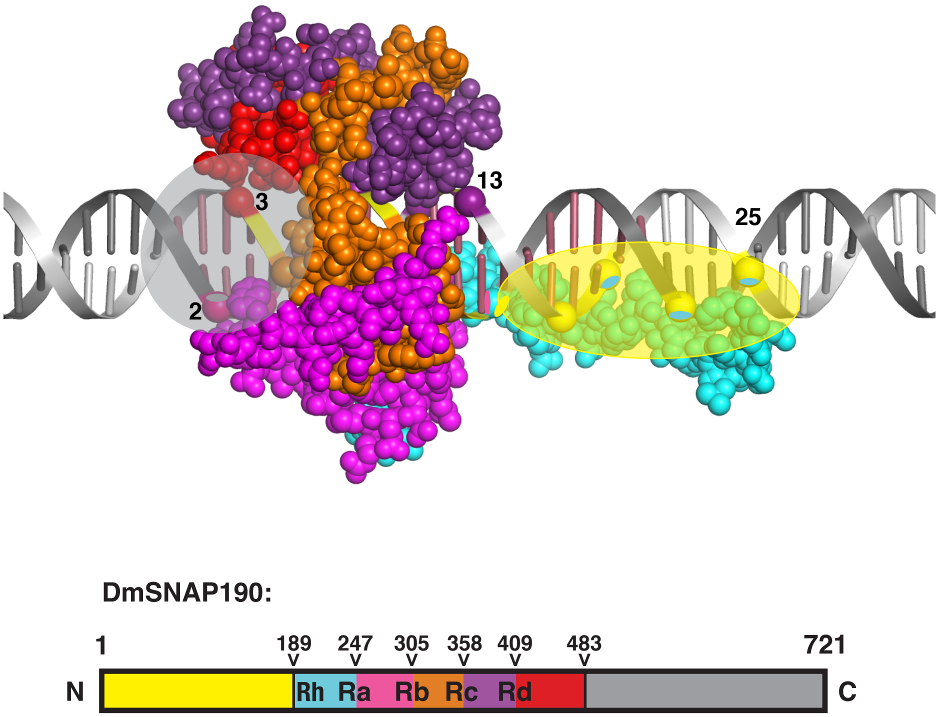

FIGURE S2. Model of DmSNAP190 bound to the U6 PSEA based upon the cross-linking data. The model was prepared as described in the text. The Myb domain is represented in space-filling mode and the N- and C-terminal domains are represented by yellow and gray ovals respectively. The colors represent the regions of the protein bounded by hydroxylamine cutting sites as indicated by the schematic diagram of DmSNAP190 shown below. DNA phosphate spheres when visible are colored as in Figure 7.
